# Medical Opinion and Movement

**Published:** 1909-11-27

**Authors:** 


### Medical Opinion and Movement.

A NEW method of inducing Local Anæsthesia in the Limbs is described in the *Lancet Clinic*. The method has been conceived and practised by Dr. J. Louis Rausdhoff, and consists in injecting a weak solution of cocaine into the main artery supplying the part to be anæsthetised, after application of an Esmarch's bandage. The following are the details of a case as reported. The patient was a man aged 72, suffering for three years from a chronic osteomyelitis of the hand which necessitated amputation. His age and condition contra-indicated a general anæsthetic. An Esmarch's bandage was applied around the arm two inches below the insertion of the deltoid muscle. Under infiltration anæsthesia the brachial artery was exposed, the needle of a hypodermic syringe inserted into its lumen, and one cubic centimetre of a two-per-cent. solution of cocaine injected into the artery in the direction of the blood current. After a lapse of two minutes the anæsthesia was complete and an amputation was carried out entirely without the knowledge of the patient.

IN cases of Fracture of the Base of the Skull the diagnostic value of blood extravasation into the spinal canal as evidenced by Lumbar Puncture is already well known. The pathology of the condition has formed the subject of some recent researches carried out by Dr. P. L. Muret and embodied in an "inaugural thesis." According to this author, the blood corpuscles of the extravasation after mixing with the cerebro-spinal fluid are precipitated into the lymphatic spaces, causing a hypertension of this fluid and inducing coma, if the extravasation be copious. Lumbar puncture at this stage gives a red fluid which escapes with a jet, and in severe cases may contain as many as a million corpuscles to the cubic millimetre. If the fluid be collected in three successive tubes from the same puncture, the number of corpuscles rapidly diminishes, showing that they tend to gravitate to the lowest part of the cord. The reaction of the nervous system consists in delirium, varying in intensity in proportion to the quantity of effused and destroyed blood corpuscles. It arises probably from two causes: mechanical irritation and possibly meningeal inflammation by the extravasated corpuscles and later intoxication of the nervous centres. The white corpuscles act as destroyers of the red. In the first stage the polynuclear are predominant, later the lymphocytes increase more and more in proportion and become the chief factor. Finally the removal of the corpuscular débris is effected by the large uninuclear elements or hæmato-macrophages. In severe cases the whole process may last a week. A rise in temperature is proportional to the number of white corpuscles. Meningeal inflammation shows itself clinically by violent headache and by hypertension of the cerebro-spinal fluid, and its presence is further shown by the fibrinous coagulum formed at the bottom of the tube. A rapid increase of lymphocytes indicates a strong reaction of the organism and offers a good prognosis. The therapeutic effect of lumbar puncture is most marked and should be repeated twice a day so long as there is an abundance of corpuscles in the fluid and marked meningeal symptoms.

HERNIA of the Bladder by traction in the course of operation for radical cure, as distinguished from true vesical hernia, forms the subject of some interesting observations by Dr. F. Brunner, of Zurich. He expresses the opinion that a large number of so-called inguino-crural cystoceles included in the statistics of vesical hernia are operative in origin and are produced in the course of operation for radical cure by the traction exercised upon the neck of the sac. This drag on the bladder in liberating the neck of the hernial sac high up is a familiar fact to surgeons. It is on freeing the sac as high up as possible on the inner side that the bladder may come into view drawn out in the form of a cul-de-sac. On this account it is important to exercise the greatest care in liberating the neck on the inner side. It is precisely in the best form of operation when the endeavour is made to follow the neck as high up as possible that the bladder is likely to be encountered. Moreover, the author points out that the vesical prolapse does not consist of a large segment in the form of a bag which would be easily recognisable, but is generally quite a short, flat projection of the bladder wall, not more than one to three centimetres long, which appear on superficial inspection to form a part with the sac. In a series of 755 radical cures for inguinal and femoral herniæ the bladder was involved 44 times—that is in 5.7 per cent. of the cases—but of these 44 cases only four were vesical hernia proper; the other 40 cases were what he terms "operative protusions." From this analysis he concludes that on an average the bladder is encountered once in 20 radical cures, and that in 10 cases in which the bladder is encountered only one case would correspond to a true vesical hernia, so that the frequency of this latter condition would be 1 in 200.

DR. C. SCHINDLER has made a special study of the automatic peristaltic movements of the uterus, and his conclusions have a bearing upon the treatment of uterine infections. His experimental researches were carried out upon rabbits, in which the movements of the uterus were observed both with the organ intact and separated from its nervous connections. He finds that in both cases the uterine contractions show the same type, so that without denying the influence that the central nervous system may exercise upon the uterus, he concludes that the uterine muscle contracts automatically by reason of the peripheral nervous centres with which it is provided. He finds, however, that there are considerable variations; that, for instance, uterine excitability is much more marked at the beginning of pregnancy and tends to diminish towards the end.

The muscular contractions of the uterine appendages, the vagina, and even the ligaments, are also automatic in character. The contractions of the tubes are of two kinds: peristaltic, that is directed towards the uterus, and antiperistaltic, towards the ovaries. Mechanical, chemical and thermal irritation excites these automatic movements. Observations in the course of abdominal operations have confirmed these antiperistaltic movements in women, and would explain the passage of infective germs upwards through the uterus and the tubes. The author points out the possibility of producing this antiperistalsis by intra-uterine medication, and the care, therefore, that should be exercised in this respect. In cases of infection, therefore, the chief indication would appear to be absolute rest of the uterus. For this purpose Dr. Schindler advocates subcutaneous injections of atropine in doses of 0.001 to 0.003 milligrammes. Prolonged hyperæmic stasis also paralyses uterine contractions, possibly by the accumulation of carbonic acid, but the stasis must be kept up for one to two hours.

---

Some interesting remarks by Dr. J. D. Rolleston on Hæmorrhagic Diphtheria are contained in the last volume of the Metropolitan Asylums Board Reports. The number of cases upon which the article is based is 78, or 5 per cent. of the total number under care. It should, however, be mentioned that cases of purpura in convalescence are excluded, as also are all those of ecchymosis about the site of injection of anti-toxin, and those of epistaxis or bleeding from the fauces alone. Moreover, some " other evidence of malignancy " is required before the case is classed as a hæmorrhagic diphtheria; in view of this rigorous selection the proportion of cases is higher than most medical men would feel inclined to expect. These cases are found principally among those patients in whom early treatment has been neglected; but exceptions occur to this rule. Hæmorrhagic diphtheria is confined to children, and its incidence is not determined by sex, season, or previous ill-health. Reaction to serum treatment is less prompt than usual, and the common sequelæ of it are much less frequent. The mortality is upwards of 80 per cent., and all patients who recover suffer from extensive paralysis. In treatment the combination of adrenalin with large doses of anti-toxic serum is recommended.

---

Mr. Lapthorn Smith, of Montreal, contributes to *International Clinics* a series of notes on gynæcological cases in which Peritonitis has arisen during the Puerperium otherwise than by Sepsis from the birth canals. In most of the cases the origin of the trouble has been in the appendix, and the author has several times been able to relieve the minds of practitioners who had called him in to treat what they took to be puerperal sepsis. It would seem also that education in Canada has proceeded so far that the relatives are only too ready to impute blame to the obstetrician in these circumstances: here it is the other way on, and puerperal sepsis is still regarded by the public far too much as an act of Providence. However, Mr. Lapthorn Smith has been able by prompt operation to save not merely medical men from undeserved opprobrium, but their patients from death also. He expresses his firm conviction that there are every year in Canada hundreds of such cases, and in the United States thousands, which are diagnosed and treated as puerperal fever and end fatally. All these patients might be saved by a timely recognition of appendix mischief and prompt removal of this organ. He very truly remarks on the great mental distress of the general practitioner in whose practice a case of septicæmia happens, and hopes that his reports of these cases may diminish this source of misery. If the cases are anything like as frequent as the author believes, it is evident that he has rendered valuable service to the community and to the profession by calling attention to them.

---

The pharmacology of a New Cardiac Tonic is very carefully described by Dr. G. D. Dawson in the *Medical Chronicle*. This is *Phytolacca abyssinica*, which contains an alkaloid and a saponin: the former of these has a deleterious effect, and it is to the saponin, or saponins, of the dried fruit that the beneficial action is attributed. The work done by the frog's heart is increased by the administration of this drug, both in the isolated ventricle and in the whole heart: probably the effect is due to direct action on the cardiac muscle. In addition, there is a quickening, brought about either by action on the auricles or on the nervous apparatus which they contain. On the blood-vessels it has a local contractile effect. In the voluntary muscles *Phytolacca* in strong solution produces an initial increase of contractility, followed by rapid weakening and loss of excitability; but motor nerves and nerve endings in voluntary muscle are not affected. In the cat and rabbit blood-pressure is increased, after a preliminary fall; but the latter is ascribed to some defect in the experimental technique. In man no gastro-intestinal irritation is caused: only two patients so far have been treated, one of whom improved distinctly, the other not. The author concludes that if further clinical trials show that the drug when given by the mouth acts as when applied directly to the tissues, it will be found a useful cardiac tonic, especially where slowing of the heart rate is undesirable. It would be of interest, also, when further research is undertaken, to ascertain the result of the active principle (a saponin) when given hypodermically.

---

In view of the foundation in London of the Radium Institute, and the sometimes extravagant hopes which this new form of treatment for malignant disease has aroused among the laity, a paper by Mr. Butlin in the *Lancet* on the results he has obtained by radium in the treatment of epithelioma and leucoplakia of the tongue is very timely. The author sums up his experiences thus: Rodent ulcers of small or moderate extent are adapted for radium treatment, which is almost painless. The results are superior to any that can be shown by operative surgery. Of advanced cases where the bone, tear ducts, etc., are attacked he has had no experience. Of four cases of epithelioma of the mouth, all amenable to surgical treatment, three got no benefit and perished miserably, but the fourth, a very early case indeed, did very well and is cured. But it is concluded that it is not yet justifiable to advise radium for any patient likely to be benefited by operation, and that there is no evidence to show that secondary growths in glands are amenable to emanations. As for leucoplakia, some failures and some partial successes are recorded. The successes are often but temporary, and so far Mr. Butlin hesitates to say whether the treatment is worth recommending. It has the great advantage of being painless and, when skilfully applied, of causing no inflammation or open sore; but its action is merely to replace the patches by scar tissue, not to restore the mucous membrane to its original condition. In view of the tuberculin boom, the author advises anyone who contemplates sending a patient abroad for radium treatment to obtain a statement in black and white that the operator believes the case to be a suitable one, not merely one for which radium "may do some good."

---

What seems to be indubitably a genuine case of Malaria contracted in England is reported by Captain Easton in the *Journal of the Royal Army Medical Corps*. The patient was a man who had never been out of England, and was, in fact, a Londoner. He was taken ill at Aldershot about three months after enlisting in an infantry regiment, and was admitted to the Cambridge Hospital with a temperature of 105° F., and a history of fever and shivering every alternate day for a week. No previous illness of the kind had ever affected him. Struck by the apparently malarial aspect of the case, the orderly medical officer, Captain Churton, took a blood film in which he found numerous rosettes. Next day the temperature was normal, but more blood films betrayed the presence of numerous benign tertian parasites. On the day after the temperature was normal in the morning and at 2 P.M. Ten grains of quinine sulphate were given in a mixture, but by 2.30 the temperature had risen to 102°, and at 6 P.M. it reached 104°. A similar dose of the mixture was then taken regularly night and morning, and no further attack occurred. The patient, when questioned, remembered being bitten on the wrist by a gnat about a fortnight before admission, when near the Basingstoke Canal. It seems probable, as the author says, that this must have been an anopheles which had derived the parasite from some malarial subject, of whom naturally there are a good number always in Aldershot.

---

The operation devised by Trendelenburg for the surgical relief of Embolism of the Pulmonary Artery has yet to meet with its first success. But in a case recorded by Krüger in the *Zentralblatt für Chirurgie*, the patient lived five days and died of preventable causes; so that it is by no means impossible that this extremely drastic operation may presently score a success, though it is unlikely ever to be practised except by very bold and very experienced surgeons. A woman was operated upon for hernia, and ten days later, when apparently on the way to an uneventful convalescence, she developed during the renewal of dressings the symptoms of a most severe pulmonary embolism. She was taken at once to the operating theatre, and Trendelenburg's operation was performed within twenty-five minutes of the first seizure. The second, third, and fourth costal cartilages were cut through and turned up towards the sternum. The lung was hooked aside, the pericardium opened, and the pulmonary artery found to be rigid and blocked completely by clot. With great difficulty, a mass of this was removed through a small incision, and even greater difficulty was encountered in suturing efficiently the wound and in stopping hæmorrhage. However, the operation was successfully concluded, and the immediate result was highly satisfactory; but, unfortunately, septic pericarditis and mediastinitis supervened, and the patient died on the fifth day. This is attributed by the author to the omission of full antiseptic precautions caused by the extreme urgency of the symptoms and the necessity for immediate operation. At the Leipzig Klinik the operation has now been performed more than half a dozen times.

---

An interesting case in which Acute Articular Rheumatism occurred in an epileptic, and in which each attack of epilepsy brought about a marked recrudescence of the incompletely-cured rheumatic affection, is reported by Minet and Herbaux in *La Province Médicale*. The authors have been unable to find reports of any exactly analogous cases in medical literature, but nevertheless the question of the relations between epilepsy and acute articular rheumatism is an interesting one. From careful analysis of the facts observed in this case, they conclude that rheumatism can have no influence on epilepsy, but, on the other hand, epilepsy would seem to have a modifying effect on the temperature curve in rheumatism, and may even have an unfavourable effect on the latter infection. In the ordinary course of events the rheumatic attack would have cleared up in about three weeks, but owing to the epilepsy causing four relapses, the case was under treatment for three months. It is well known that an attack of rheumatism is often brought on by fatigue and over-exertion, and it is possible that the unfavourable influence of epilepsy on rheumatism can be explained by the fatigue engendered by the epileptic attack lighting up afresh the half-cured rheumatic infection. Though this explanation is rational, it must be remembered that little is understood of the true nature of epilepsy and rheumatism. We are gradually learning something of their symptomatology, but their exact ætiology is unknown. It is therefore only possible in the case under discussion to have recourse to more or less ingenious hypotheses, which are incapable of proof and verification.

